# The Effect of Pediatric Colorectal Short-Term Medical Service Trips on Self-Reported Confidence in Patient Care in Volunteers in the Home Country

**DOI:** 10.5334/aogh.2744

**Published:** 2020-03-12

**Authors:** Wilfried Krois, Peter Gröpel, Pastora X. Hernandez, Juan Craniotis-Rios, Martin L. Metzelder, Richard J. Wood, Marc A. Levitt, Carlos A. Reck-Burneo

**Affiliations:** 1 Division of Surgery, Clinical Department of Pediatric Surgery, Medical University of Vienna, Vienna, AT; 2 Division of Sport Psychology, Department of Sport Science, University of Vienna, AT; 3 Hospital Ruth Paz, San Pedro Sula, HN; 4 Center for Colorectal and Pelvic Reconstruction, Nationwide Children’s Hospital, Columbus, Ohio, US; 5 Division of Pediatric Surgery, Children’s National Hospital, Washington, D.C., US

## Abstract

**Introduction:**

Short-term international medical service trips (MST) are established means to access specialized medical aid in resource-limited areas. The field of pediatric colorectal surgery is a subspecialty in pediatric surgery that mainly treats anorectal malformations (ARM) and Hirschsprung disease (HD). This study aimed to investigate the impact of MST on the donors’ perception of competency concomitantly to the impact on patients in the donors’ home country. We also wanted to investigate whether the donors’ pre-existing experience in the field of ARM and HD affects the experience they gain during the MST, and the subjective perception in treating patients in their base country.

**Methods:**

We created a questionnaire for the international medical staff participating in MSTs on the unique topic of pediatric colorectal diseases. The questionnaire was split into three parts: essential experience (1) in the field of colorectal surgery of the participant, the experience and impact on patient care in the home country during and after the MST in ARM (2), and in HD (3).

**Results:**

We collected data from 20 participants (6 female, 14 male). The majority of them had prior experience with the MST program (75%) and came from institutions specialized in the treatment of pediatric colorectal disorders (80%). Participants felt that MST improved patient care in both the host country (p < 0.001) and their home country (p < 0.001). Experienced and less experienced participants did not differ in the overall MST evaluation (ps > 0.08). They reported that their competencies to treat ARM and HD improved significantly in response to the MST (ps < 0.001). Improvements in ARM and HD treatment were associated with the number of supervised HD surgeries during MST, while the other forms of participation were unrelated to the improvements.

**Conclusion:**

The results of our questionnaire indicate that participation in MST in the specialized field of pediatric colorectal surgery helps to improve confidence in the care and treatment of affected patients in both the host and donor countries, independent of previous surgical experience.

## Introduction

Short-term medical service trips (MST) are an established form of access to medical aid in resource-limited areas in lower and lower-middle-income countries (LMICs), especially in African and Central American regions [1,2]. Due to the scarcity of medical care in LIMCs, there seems to be a need for upscaling pediatric surgical capacity to address significant surgical delay, surgical backlog, and unmet needs [3]. However, there are concerns about cost-effectiveness, continuity of care, and a sparsity of data about social, economic and diplomatic consequences. Nevertheless, in recent years, improvements to organizational structures and logistics have been achieved by various MST with different thematic fields of pediatric surgical expertise. The aim is to improve efficiency and outcomes [4,5]. Surgeons volunteer for the trips to train and improve their personal skills on socially disadvantaged patients in LIMCs, but questions and concerns exist about them operating without supervision and adequate training [2,6].

On the other hand, high surgical volume seems to be linked to improved outcomes accompanied by an arising discussion about the dilemma in treatment of rare congenital malformations [7,8]. “Karate is like boiling water: without heat, it returns to its tepid state” is a wisdom by Gichin Funakoshi, the founder of modern karate [9]. It can also be applied to the current pediatric surgery scenario: less exposure to rare diseases is caused by the increasing number of pediatric surgeons finishing fellowship, and with fewer cases, leading to less competency is acquired in its repair [7]. New concepts such as centralization, specialization, team approaches, simulations and regular participation in MST with specific thematic programs seem to be possible options to counteract decreasing competency in their own country. A team approach may help to maintain exposure to adequate index case volumes, especially in geographically isolated practices or low-volume departments in high-income countries [8]. Considering that residents perceived benefits regarding cultural competency, communication skills, adaptability, and desire for service years after their involvement [10], regular participation in specialized MST seems to have beneficial aspects.

The field of pediatric colorectal surgery for the two main groups of colorectal problems, anorectal malformations (ARM) and Hirschsprung disease (HD), is a subspecialty in pediatric surgery that requires skills and training to maintain competency. With a worldwide incidence of about 4.1 per 10,000 in ARM and 1.86 per 10,000 born alive with HD [11,12], centralization and specialization seem to be ways to improve results and outcomes for affected children in high-income countries (HICs) as well. Under this premise, pediatric surgeons from HICs volunteering on a periodical basis to support local staff in LMICs to treat and operate on children with complex ARM and HD seems to be beneficial for patients in both host and donor countries.

Several groups report outcomes after MSTs for patients, but less is known about whether MSTs also affect donors’ competency and patient care in the donor country. Two international working teams, led by some of the authors, have organized MSTs in the special field of pediatric colorectal diseases for several years, with long-term partnerships mainly in Central and South American LMICs. The aim of this study was to investigate the impact of MSTs organized by the team on the donors’ feeling of competency and the impact on the patients in the donors’ country. We also wanted to investigate whether the donors’ pre-existing experience in the field of ARM and HD surgery affects the experience they gain during the MST, and the subjective perception in treating patients in the participating donor country.

## Methods

We created a questionnaire on the special topic of pediatric colorectal diseases (see Appendix). Medical staff participating in the MSTs organized by two groups of international working members were asked to compete the questionnaire. The two groups are “Colorectal Team Overseas”, led by American pediatric surgeons who started MSTs on a regular basis, and “Helping Hands for Anorectal Malformations International”, led by Austrian pediatric surgeons who began annual MSTs in 2012. These two teams have similar and partly overlapping activities, concentrating on the treatment of patients with ARM and HD in Central America (mainly Honduras) as well as South America. At the time of publication, “Colorectal Team Overseas” had performed 22 trips and “Helping Hands for Anorectal Malformations International” had completed six trips. All of the MSTs were organized in close collaboration with local pediatric surgeons. In each trip, at least one fellowship trained expert in pediatric colorectal and pelvic reconstructive surgery was established as the clinical team leader. The postoperative patient care and follow-up was performed by the participating local team members. The same local teams have been present during the MSTs and have been trained to perform this type of surgeries on their own. From year to year, surgery complexity increased, as less demanding cases were operated by the native teams. All participating volunteers paid all expenses themselves and operated free of charge.

In our questionnaire, we included permanent and temporary medical staff from both teams (“Colorectal Team Overseas” and “Helping Hands for Anorectal Malformations International”) who had participated in previous MSTs. The questionnaire was split into three parts: Part I investigated the basic experience in the field of colorectal surgery of the participant, Part II investigated the experience and impact on patient care in the home country during and after the MST in ARM, and Part III was on the topic of HD. For data collection, we used the RedCap tool.

Statistical analyses were performed using the SPSS statistics software. One-sample *t* tests were performed to test whether participants’ subjective experience with MST and their feelings of competency to treat ARM and HD were different from 3 (= the scale median). Spearman’s correlation coefficients (rho) were calculated to test which forms of participation in ARD and HD treatment (assisted at, performed under supervision, performed without supervision, supervised) were related to participants’ feelings of competency. Hierarchical multiple regression analyses were conducted on competencies in ARM and HD treatment to test whether MST had different benefits for more and less experienced participants. The number of ARM and HD surgeries at home clinic, respectively, was controlled for at the first step, the number of ARM and HD surgeries during MST and participants’ years of experience were entered as the second step, and their interaction term was entered as the third. In all analyses, predictor variables were standardized before their interaction term was calculated. The level of significance was set at *p* ≤ 0.05 (two-tailed).

## Results

Data from 20 participants (6 female, 14 male) were collected. The majority of participants had prior experience with the MST program (75%) and came from institutions that specialized in the treatment of pediatric colorectal disorders (80%). Participants’ characteristics are presented in Table 1. In general, participants felt that the MST would improve patient care in both the host country, *M* = 4.85, *t*(19) = 22.58, *p* < 0.001, and their home country, *M* = 4.50, *t*(19) = 8.11, *p* < 0.001. They also stated that they would like to attend another MST program in the future; this especially applied to MSTs with a focus on pediatric colorectal disorders, *M* = 4.95, *t*(19) = 39.00, *p* < 0.001, but also to MSTs focusing on other conditions than pediatric colorectal disorders, *M* = 4.05, *t*(18) = 3.39, *p* = 0.003. Experienced and less experienced participants did not differ in the overall MST evaluation (*p*s > 0.08).

**Table 1:** Participants’ characteristics.

	*N*	(%)

**Sex**		
Female	6	(30)
Male	14	(70)
**Age**		
31–40 years	8	(40)
41–50 years	8	(40)
51–60 years	4	(20)
**Home country**		
Austria	4	(20)
Germany	1	(5)
Honduras	1	(5)
Peru	2	(10)
South Africa	2	(10)
Switzerland	1	(5)
USA	9	(45)
**Home clinic specialized in the treatment of pediatric colorectal disorders**		
Yes	16	(80)
No	4	(20)
**Experience**		
Resident <3 years	0	(0)
Resident 3–5 years	1	(5)
Resident >5 years	2	(10)
Specialist <5 years	6	(30)
Specialist 6–10 years	4	(20)
Specialist 11–15 years	2	(10)
Specialist >16 years	4	(20)
Not reported	1	(5)
**Prior short term medical mission trips**		
0	5	(25)
1–3	3	(15)
4–9	9	(45)
>10	3	(15)

### Anorectal malformations experience

**Figure 1:**
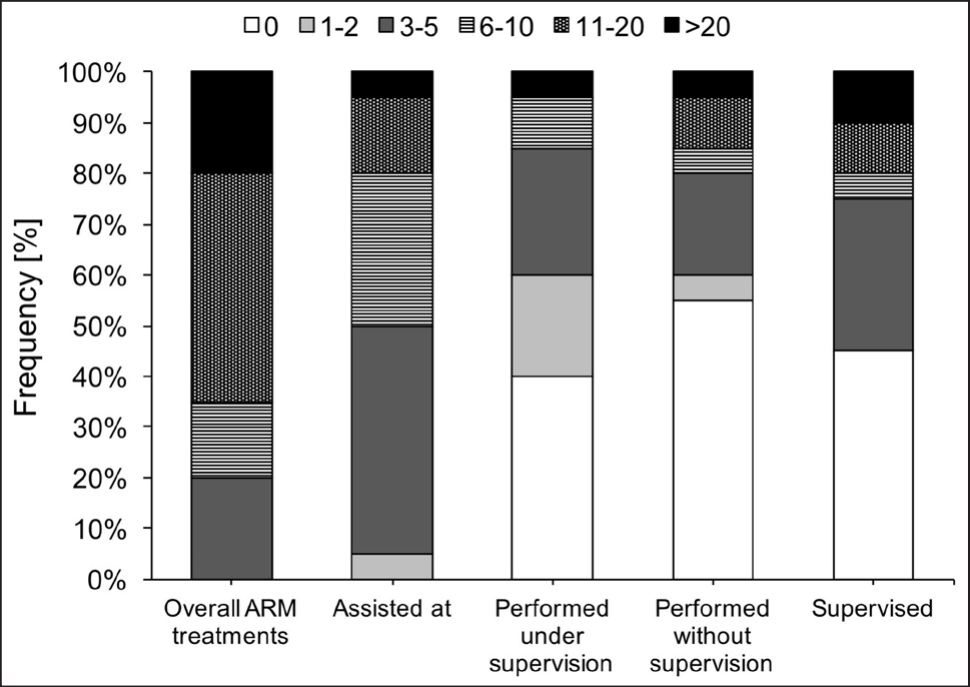
Participation in ARM treatment during MST.

During the MST program, participants were involved in multiple ARM treatments (*Mdn* = 11 to 20 times), in which they either assisted in an ARM surgery, performed the surgery themselves (with or without supervision), or supervised the surgery (Figure 1). Participants reported that their competency to treat ARM improved significantly in response to the MST, *M* = 4.04, *t*(19) = 5.88, *p* < 0.001. This improvement was positively associated with the number of ARM surgeries performed under supervision, Spearman’s rho = 0.53, *p* = 0.02, while the other forms of participation did not show any significant effect. The relationship between the number of supervised surgeries and competency improvement remained significant even after controlling for the number of participants’ supervised ARM surgeries at their home clinics (Table 2). Experienced and less experienced participants did not differ in self-reported competency improvement in ARM treatment (*p* = 0.66), and benefited equally from the supervision during the MST as indicated by the non-significant Expertise × MST interaction (Table 2).

**Table 2:** Multiple regression analysis of supervised ARM surgeries at home clinic and during MST on self-reported competency in ARM treatment.

Condition	*B*	*SEB*	β	*R^2^*	Δ*R^2^*

Step 1				0.17	
Home clinic	0.39	0.22	0.41		
Step 2				0.65**	0.48**
Home clinic	–0.07	0.18	–0.07		
MST	0.81	0.18	0.84**		
Expertise	0.05	0.15	0.05		
Step 3				0.71**	0.06
Home clinic	0.08	0.19	0.08		
MST	0.86	0.17	0.89**		
Expertise	0.04	0.14	0.04		
Expertise × MST	–0.32	0.18	–0.31		

*Note: B* = unstandardized coefficients; *SEB* = standard errors of unstandardized coefficients for the variables in the final regression equation; β = standardized coefficients; *R^2^* (Δ*R^2^*) = cumulative (change in) variance accounted for at each step. The number of supervised ARM surgeries at home clinic is controlled for at Step 1. ** *p* ≤ 0.01.

### Hirschsprung disease experience

**Figure 2:**
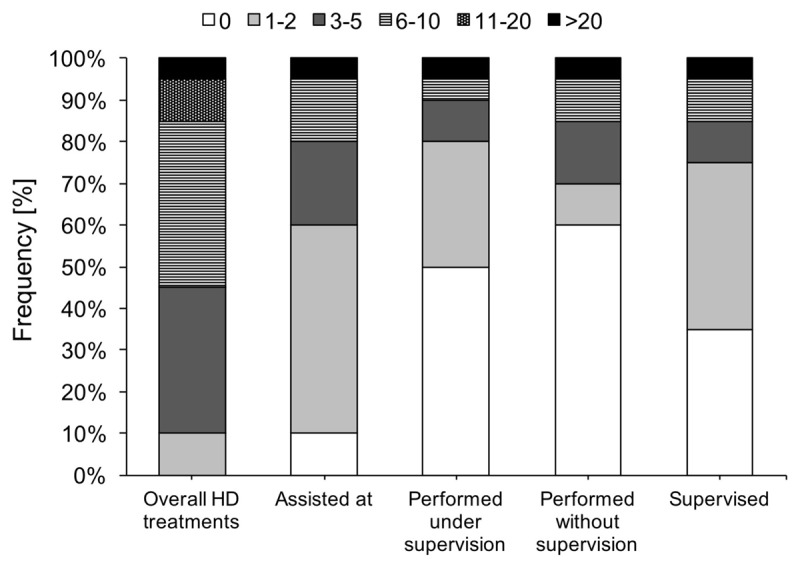
Participation in HD treatment during MST.

Similar to ARM experience, participants were involved in several HD treatments (*Mdn* = 6 to 10 times), in which they either assisted in a HD surgery, performed the surgery themselves (with or without supervision), or supervised the surgery (Figure 2). Participants felt that their competency in HD treatment improved after the MST, *M* = 3.93, *t*(19) = 4.88, *p* < 0.001. Again, this improvement was only associated with the number of supervised HD surgeries, Spearman’s rho = 0.59, *p* = 0.007, while the other forms of participation were unrelated to the self-reported competency improvement. The relationship between the number of supervised HD surgeries and competency improvement turned to non-significant when controlled for the number of participants’ supervised HD surgeries at their home clinics (Table 3). Experienced and less experienced participants did not differ in self-reported competency improvement in HD treatment (*p* = 0.36).

**Table 3:** Multiple regression analysis of supervised HD surgeries at home clinic and during MST on self-reported competency in HD treatment.

Condition	*B*	*SEB*	β	*R^2^*	Δ*R^2^*

Step 1				0.19	
Home clinic	0.42	0.21	0.44		
Step 2				0.40*	0.20
Home clinic	0.07	0.26	0.08		
MST	0.52	0.26	0.54		
Expertise	0.15	0.20	0.15		
Step 3				0.42	0.03
Home clinic	0.15	0.27	0.15		
MST	0.56	0.27	0.58		
Expertise	0.14	0.20	0.15		
Expertise × MST	–0.19	0.25	–0.19		

*Note: B* = unstandardized coefficients; *SEB* = standard errors of unstandardized coefficients for the variables in the final regression equation; β = standardized coefficients; *R^2^* (Δ*R^2^*) = cumulative (change in) variance accounted for at each step. The number of supervised HD surgeries at home clinic is controlled for at Step 1. * *p* ≤ 0.05.

## Conclusion

The results of our questionnaire indicate that the participation in MST in the special field of colorectal pediatric surgery helps to improve confidence in care and treatment of affected patients in both the host and donor country, independent of previous surgical experience.

As a matter of course, at least one senior supervisor with high experience in the field of pediatric colorectal surgery was present to supervise all operations and treatments in the pediatric colorectal MSTs organized by our organizations. In all trips, at least one or two attendants who were specialists with a minimum of five years’ experience were involved in all surgeries and residents never performed surgery unsupervised. The number of ARM and HD patients treated during one MST could easily exceed the number of patients born with the disease during one year in the donor country. Even though the supervisor was working in a specialized clinic for pediatric colorectal surgery, the benefit was identical in the less experienced group of participants. The number of patients consolidated in the short time period of the trip seems to boost the experience and self-reported competence in all participants.

Results from regression analyses indicate that MST has a positive effect on participants’ feelings of competency over and above the usual supervised practice at their home clinic. Moreover, the benefits of MST are not limited to less experienced donors (residents). Even participants who have been working in the field for many years may benefit from MST.

In our results, the benefit and increase in confidence in treating patients with ARM was higher compared with the benefit in treating patients with HD. A reasonable explanation for these findings might be that the number of patients treated with HD was lower than patients with an ARM in the MST, which might have weakened the beneficial effect.

As reported by other groups [10], the experience of the volunteers was positive and they stated that they would like to attend a MST program for pediatric colorectal surgery, as well as other thematic programs, in the future. Three quarters of our volunteers repeatedly attended in MST, and all participants felt that it would help to improve patient care in both the host and the donor country.

These findings might help to support social and economic co-operations between LMICs and HICs in the future, as the benefit for both sides in regard to optimizing patient care also in HICs in times of maintaining competency concerns was demonstrated.

## Additional File

The additional file for this article can be found as follows:

10.5334/aogh.2744.s1 Data. Dataset of returned questionnaires.

## Figures and Tables

**Table:** 

MST	Short-term international medical service trip
ARM	Anorectal malformation
HD	Hirschsprung disease
SD	Standard deviation
LMICs	Lower and lower-middle-income countries
HICs	High-income countries
HH	Helping Hands for Anorectal Malformations International (organization)
CTO	Colorectal Team Overseas (organization)
